# P-2267. Autopsies: Revisiting the Gold Standard

**DOI:** 10.1093/ofid/ofae631.2420

**Published:** 2025-01-29

**Authors:** Helen Tsai, Grace Y Minamoto, Daniel A Burack, Rithva Ramesh

**Affiliations:** Montefiore Medical Center, Bronx, New York; Montefiore Medical Center, Bronx, New York; Montefiore Medical Center, Bronx, New York; Albert Einstein College of Medicine, Bronx, New York

## Abstract

**Background:**

Infections in solid organ transplant (SOT) recipients continue to be a clinical challenge despite modern diagnostics. Autopsies have the potential to identify missed infectious diagnoses thereby informing the practice of transplant infectious diseases. We sought to evaluate the clinical value of autopsies by analyzing pre-and post-mortem infectious diagnoses of deceased SOT recipients.

**Figure:**
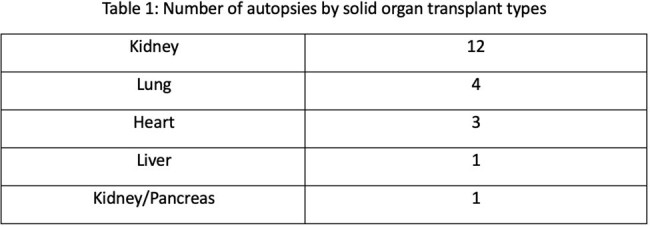

**Methods:**

We retrospectively reviewed deceased adult SOT recipients who underwent autopsies at our institution from January 2017 to December 2022. Electronic medical chart review of these recipients was conducted to determine the clinical infectious diagnoses during the hospitalization in which death occurred. Autopsy reports were reviewed for post-mortem pathological findings. Patients who expired near admission without clinical information or did not expire from infection were excluded. Two physicians independently reviewed clinical information and pathological data and applied the Modified Goldman Criteria to categorize clinical and autopsy infectious discrepancies.

**Results:**

During the study period, 21 autopsies were performed (Table 1). Pre-mortem infectious diagnoses included bloodstream infections (n = 11), pneumonia alone including 1 case of *Rhizopus* and 1 case of *Pneumocystis* (n = 7), CMV viremia (n = 2), cholangitis (n = 1), and sepsis of undetermined etiology (n = 2). In no instances did an autopsy exam reveal an infection undiagnosed pre-mortem, and thus there were no diagnostic errors that met Modified Goldman Criteria. No specific histopathological staining was performed in the cases with CMV viremia or suspected *Pneumocystis* pneumonia. In the case of *Rhizopus*, no comment regarding evidence of angioinvasion or extent of infection was made in the autopsy report.

**Conclusion:**

We did not identify any missed infectious diagnoses on post-mortem evaluation that would have impacted management or outcome. These findings emphasize the importance of collaboration between infectious disease specialists and anatomic pathologists to direct histopathologic and microbiologic testing on tissues of clinical interest. Accurate autopsy diagnoses for SOT recipients, who are at risk for opportunistic and emerging infections, can critically advance transplant medicine.

**Disclosures:**

All Authors: No reported disclosures

